# Anchor-free real-time vehicle tracking using LiDAR data

**DOI:** 10.1371/journal.pone.0323287

**Published:** 2025-05-09

**Authors:** Yibin Zhang, Qiyang Luo, Shuichao Zhang, Hongchao Liu, Siyu Xie

**Affiliations:** 1 School of Civil and Transportation Engineering, Ningbo University of Technology, Ningbo, Zhejiang, China; 2 Zhejiang Engineering Research Center of Digital Road Construction Technology, Ningbo, Zhejiang, China; 3 Department of Civil, Environment, and Construction Engineering, Texas Tech University, Broad-way Lubbock, Texas, United States of America; 4 Faculty of Science Engineering, University of Nottingham Ningbo, Ningbo, Zhejiang, China; Tsinghua University, CHINA

## Abstract

Roadside LiDAR systems can generate real-time microscopic vehicle trajectories applicable to develop intelligent transportation systems and aid the operations of connected and autonomous vehicles. Tracking is the most crucial data processing step to generate accurate and reliable trajectories of road users from raw point clouds collected from LiDAR sensors. In this paper, a new tracking mechanism is proposed for real-time tracking, which is based on the 2D LiDAR data structure with the Simple Online and Real-Time Tracking (SORT) algorithm. The traditional method of using bounding boxes to identify vehicles is replaced by center points of vehicles inspired by track-by-point approach. The developed index that integrates the distance between the LiDAR serves as a more accurate way of defining the spatial location of vehicles. The proposed methodology was evaluated using data collected by a 32-channel portable LiDAR at three signalized intersections. The results showed that the proposed method has a higher tracking accuracy and a faster computation speed compared to the traditional bounding box approach, indicating improvement toward real-time applications. *Index Terms*—Real-time tracking; Roadside LiDAR; Trajectory; Track by point; SORT.

## 1. Introduction

Microscopic vehicle trajectory data refers to the movement and position data of individual vehicles at a very fine temporal and spatial resolution. The applications of microscopic vehicle trajectory data are numerous and diverse. In the field of traffic operations, it can be used to analyze traffic flow patterns and congestion [1]. optimize traffic control systems [2], and improve the design of road network. Likewise, this data can also help improve traffic safety by analyzing crash patterns and contributing factors through the analysis of vehicle movement before and during crashes. The resulting insights can lead to targeted interventions such as road design modification, speed control, and safe driving promotion. Additionally, microscopic vehicle trajectory data can aid the development of connected and autonomous vehicle environments by providing detailed insights into the movement patterns and behaviors of vehicles, which can be used to improve vehicle control systems [3], optimize traffic flow [4], and real-time evaluation of traffic safety [5]. Specifically, Benjamin Coifman [6] believes that microscopic vehicle trajectories are intended to help advance traffic flow theory in general and car-following models in particular [7]. However, the historical standard for vehicle trajectory data does not provide the necessary precision [8]. Overall, microscopic vehicle trajectory data is a valuable resource for researchers and practitioners in a variety of fields, as it provides a detailed and accurate picture of vehicle movement and behavior.

Advancements in sensor technologies have significantly improved the collection of microscopic vehicle trajectory data, with video cameras, radar, and LiDAR being the most commonly used options [9]. Among these, LiDAR data poses the greatest challenge for real-time processing due to its large size and high computational requirements, while video data benefits from established compression techniques but struggles with privacy concerns and reduced performance in low-light conditions [10]. Radar data, although compact, requires complex algorithms and lacks precision for accurate trajectory tracking [11]. LiDAR sensors, available as on-board or roadside systems, offer key advantages such as precise vehicle localization, strong performance in low-light conditions, and privacy protection [12,13]. Compared to on-board, roadside LiDAR systems generate real-time vehicle trajectories from point cloud data, helping to address data gaps for non-connected users and providing valuable insights for traffic management and safety monitoring in mixed traffic environments [14].

Background filtering, object clustering, object classification, and object tracking are the four major steps for processing LiDAR data [15]. Initially, background filtering is conducted to remove the stationary ground points, unwanted noise, and background objects such as trees, buildings, traffic lights, and other roadside objects [16], which is followed by point clustering to group the remaining mobile objects together individually [17]. Then, each cluster is classified either as a vehicle, pedestrian, or any other types of road user such as bicyclists, skateboarders depending on variables like vertical and horizontal point distribution, distance to the LiDAR, and inclination angle of the cluster fitting plane etc. [18,19]. Finally, each classified road user is tracked across multiple consecutive frames of point cloud data to obtain the vehicle trajectories. Object tracking is the last and the most important step in LiDAR data processing. Its accuracy directly affects the reliability and credibility of the trajectories for application. Numerous models and algorithms have been proposed by researchers to enhance tracking accuracy. These include Graph Neural Network (GNN)[14], Joint Probabilistic Data Association (JPDA) [20], Multiple Hypothesis Tracking (MTH) [21], and Hungarian algorithms [22]. GNN uses the Euclidean distance to associate the nearest vehicle within different point cloud frames. It is simple to implement and has relatively low computational load [18,23]. For instance, Zhao [24] adopted the GNN to track vehicles and pedestrians, achieving a tracking accuracy of about 95%. However, GNN can lead to false tracking and missing data points, particularly in dense traffic scenarios. To address this issue, Zhang applied JPDA in a complex traffic environment, resulting in improved tracking performance. Nonetheless, JPDA necessitates significant computational resources and is susceptible to combinatorial explosion. Furthermore, real-time applications based on current methods are still challenging due to the difficulties in obtaining consistent tracking accuracy and computational complexity. Hence, it is crucial to develop and test other real-time tracking algorithms that focus on tracking accuracy, universality, and computation time to leverage benefits from the highly accurate 3D point clouds obtained from roadside LiDAR systems.

Theoretically, tracking can be treated as the problem of multiple object tracking (MOT). The MOT problem can be viewed as a data association problem where the aim is to associate detections across frames in a consecutive sequence based on the geometry and motion of the object being tracked [25,26]. Two major categories of MOT are Detection-Based Tracking (DBT) and Detection-Free Tracking (DFT), depending on how objects are initialized [27]. DBT, also known as “tracking-by-detection”, first detects objects and then links them into trajectories. In contrast, DFT requires manual initialization of a fixed number of objects in the first frame. Consequently, DBT is more widely used since new objects are discovered and disappearing objects are automatically terminated. Multiple frameworks exist within DBT, such as Joint Detection and Embedding (JDE), Center Track [28,29], which process detection and association jointly or separately. Simple online and real-time tracking (SORT) is another DBT based tracking method employed in image-based tracking that uses a much simpler framework[30]. SORT performs Kalman filtering [38] in image space and frame-by-frame data association using the Hungarian method using an association metric that measures bounding box overlap [31]. Recently, in addition to various adaptations of the DBT algorithm [32–34], with the growing interest in the attention mechanism of language models, the Transformer architecture has garnered significant attention and has been successfully applied across various fields, including object tracking [35–37]. This architecture supplants both the detection model and the location prediction model in the DBT paradigm [38]. Specifically, MeMOT [39] has employed a memory buffer within the model to leverage both short-term and long-term information for enhanced inference capabilities. Teng Fu has developed DeNoising-MOT [40], a transformer-based approach, to address challenges arising from severe occlusions. However, one of the biggest downsides of point-cloud data is that it does not convey any scene semantic information. Therefore, performing the supervised tracking methods on LiDAR data requires additional steps.

Existing tracking methods often rely on bounding boxes to express motion model states and use Intersection-over-Union (IoU) as an association metric. However, these approaches have notable limitations. First, the detection performance relies heavily on the hyperparameters of the anchors, such as the size, aspect ratio and number of anchors [41]. Second, a larger anchor box is needed to ensure sufficient overlap with the boxes representing the ground truth [42]. To address these challenges, anchor-free methods like CenterTrack, which utilize point-based features [43,44] to infer object motion, have emerged as a promising alternative. By simplifying object association across frames, these methods offer better adaptability to road user variability and reduce reliance on complex bounding box configurations. Inspired by these developments, this study builds upon the SORT algorithm and introduces a novel tracking mechanism tailored for roadside LiDAR systems. The proposed method replaces bounding boxes with the center points of vehicles, a key principle of the track-by-point approach. Additionally, a new index that incorporates the distance between the LiDAR and the vehicle (DbLV) is developed to enhance spatial localization accuracy. By leveraging the 2D LiDAR data structure, the method integrates a Kalman filter for motion prediction and applies the Hungarian algorithm for one-to-one bipartite matching, ensuring efficient and accurate tracking.

Overall, the objective of this paper is to introduce a new real-time tracking methodology as a part of point cloud data processing to collect road user trajectories in real-time using roadside LiDAR sensors. This methodology includes three novel features to improve the efficiency and accuracy of the tracking process:

- Using a 2D data structure that utilizes azimuth and vertical angle as its basis, derived from 3D point cloud data, to enhance computational efficiency.
- A new strategy that could identify the stopping vehicles is used to get accurate background results.
- Based on the center point of vehicles, implementing an index that integrates (i) the gap between the central point of vehicles and (ii) the distance from the vehicle to the LiDAR for tracking instead of the commonly used intersection-over-union (IoU) method for bounding boxes.

The rest of the paper is organized as follows: Section II presents the framework of the proposed methodology with detailed information about the index used. Section III presents the results of a case study conducted to test the methodology. The final section summarizes the findings from this study along with its limitations and perspectives on future work.

## 2. Methodology

**Fig 1:**
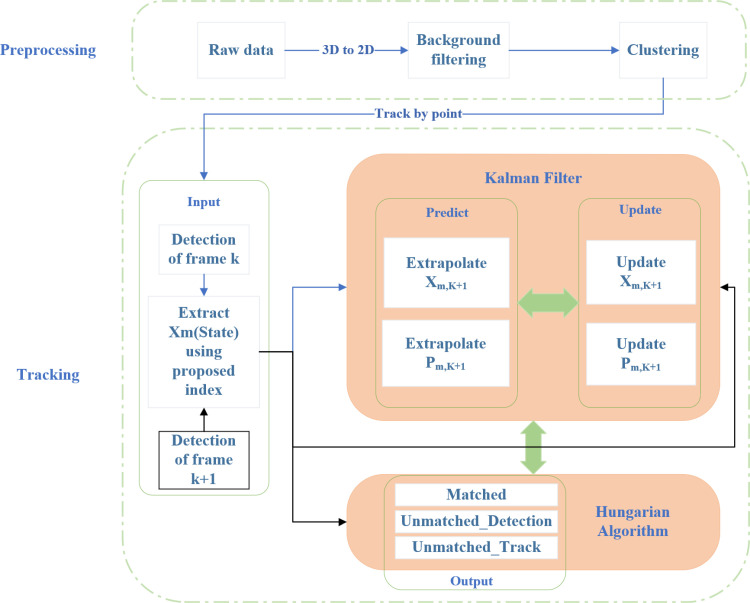
Framework of the Methodology.

In this study, a three staged methodology is used to track objects at a signalized intersection: (i) Background filtering; (ii) Object detection; (iii) Object tracking. As mentioned earlier, the main focus of this study is on object tracking, which is performed by estimating an object’s state and data association between two successive frames using a novel index. Fig 1 shows the workflow summarizing the framework of the methodology. A detailed explanation of the procedures is provided in the following section.

### 2.1 Background filtering

As the subsequent steps mostly rely on the total number of points, background filtering acts as a crucial preprocessing step not only for improving accuracy but also for faster computation speed. The purpose of this step is to get reference background data (such as trees, noises, buildings) that can be used to filter the incoming frames.

Currently, the 3D-density-statistics-background-filtering (3D-DSF) is commonly used for background filtering. It involves calculating the density of points in different regions of the 3D space, identifying high-density areas that likely contain relevant objects and low-density areas considered as background noise. Despite its simplicity, the 3D-DSF has two major downsides. The first is that it requires significantly higher computation time when the data size increases, and the next one is that it can’t identify the stopping vehicles. To overcome these problems, a faster and accurate method to filter background points is used in this study.

One outstanding feature of roadside LiDAR is that most data points are background points, which means 1) in a static frame, only a few data points belong to moving object. 2) for consecutive frames, the DbLV vibration of specific laser in each subspace caused by moving objects is rare. Based on these two features, firstly, the DbLV and z coordinate are used to select the region of interest. As shown in Table 1, the Counts is the total number of data points in one frame. 75% of the points are within 55 meters of the LiDAR and below 0m vertically (z-coordinate).

**Table 1.** Statistics of DbLV and z Coordinate of Raw Data.

	DbLV(m)	z coordinate(m)
Counts	43769	
Mean	38.288	-1.010
Min	2.012	-10.603
25%	9.588	-2.275
50%	25.832	-1.096
75%	54.384	0.000
Max	216.388	15.462

Based on these statistics, it is feasible to eliminate certain data points by defining a region of interest, given that the height of a vehicle cannot exceed 15.462 meters. Therefore, 80m for DbLV and a vertical range of -4m to 1m for the z-coordinate are selected. This decision represents a compromise between maintaining accuracy and removing redundant data points. Secondly, the most common value for each laser in each subspace is recognized as background.

**Fig 2:**
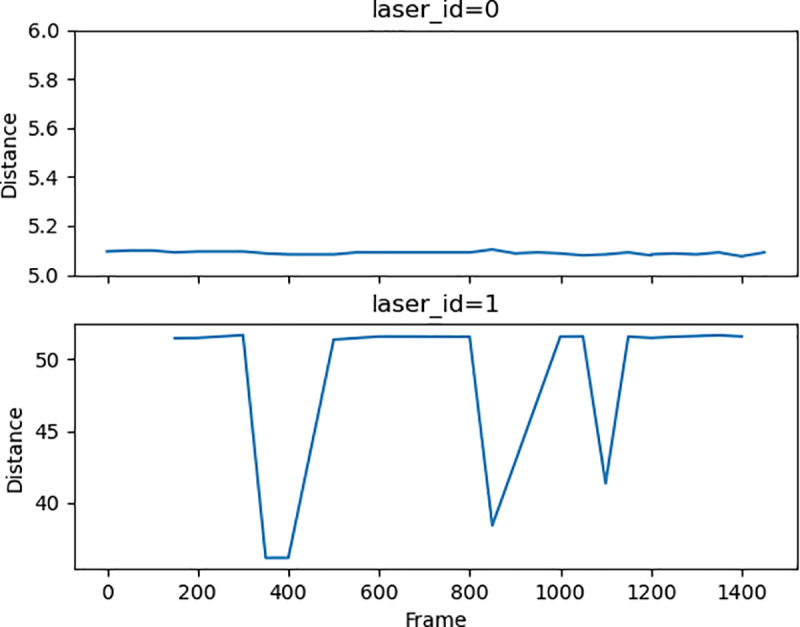
DbLV of Two Lasers in Same Azimuth for 1500 Frames.

For example, Fig 2 shows the DbLV (distance) of 2 laser IDs in same subspace (azimuth 0) for sampled frames from total 1500 frames. The DbLV keeps constant around 5.1m for laser ID 0 indicates 5.1m for this subspace is definitely a background point. Although DbLV fluctuates over frames for laser ID 1, the most common value can be defined as a background point value. This is because the duration (frames) of vehicles occupies a specific subspace is significantly shorter compared to background points. The background data can be obtained for each laser in each azimuth, then all incoming frames can be filtered based on the background data. Furthermore, since identifying background points can be accomplished offline, filtering the incoming frames can be carried out in real-time without difficulty. With this approach, unlike the DBSCAN-based algorithm, the stopped vehicles waiting for traffic lights can be identified.

### 2.2 Object detection

After background filtering, the remaining points can be grouped into different objects using clustering techniques. The authors proposed a clustering methodology combining the region growing algorithm with counted component labelling in their previous study, which is adopted in this study as well.

The traditional study used density based spatial clustering applications with noise (DBCSAN) to cluster the 3D data point. The principle of DBSCAN is that if the number of data points within a searching area is greater than or equal to a predefined MinPts (Min of points number) value, those data points will be clustered to form an object. Nonetheless, DBSCAN can encounter time-related issues when handling larger datasets and may require predefining parameters for different regions.

The clustering method adopted in this paper processes the data in the 2D structure and keeps the parameters for the whole area. The principle of the clustering method is to search the neighbors of the seed point to grow its region until no satisfied neighbors are found, then it goes to next seed point.

### 2.3 Object tracking

#### 2.3.1 Multiple object tracking principle.

Luo [45] summarized that the objective of multiple object tracking is to find the “optimal” sequential states of all the objects, which can be generally modeled by performing MAP (Maximum a posteriori) estimation from the conditional distribution of the sequential states given all the observations:


$${\hat{S}}_{1:t}=\underset{S_{1:t}}{argmax}P(S_{1:t}|O_{1:t})$$ (1)

where $S_{1:t}=\left\{S_{1},S_{2},\ldots ,S_{t}\right\}$ denotes the sequential states of all the objects from the first frame to the *t*-th frame, and $O_{1:t}=\left\{O_{1},O_{2},\ldots ,O_{t}\right\}$ represents the collec*t*ed sequential observations of all the objects from the first frame to the *t*-th frame. ${\hat{S}}_{1:t}$ is the estimated optimal match between $S_{1:t}$ and $O_{1:t}$. One way *t*o solve this problem is conversely minimizing an energy function $E(S_{1:t}|O_{1:t})$:


$${\hat{S}}_{1:t}=\underset{S_{1:t}}{argmax}\exp\left({-E(S}_{1:t}|O_{1:t}\right))/Z=\underset{S_{1:t}}{argmin}E(S_{1:t}|O_{1:t})$$ (2)

where Z is a normalization factor to ensure $P(S_{1:t}|O_{1:t})$ to be a probability distribution.

#### 2.3.2 State estimation using kalman filtering.

After clustering, the centroid of each cluster (vehicle) can be extracted as a reference point to represent the vehicle’s position in the current frame and further used to calculate DbLV. Using this information, the state of each target at an instant can be represent-ed in the form of the following index:


$$x={[u,v,d,\;\dot{u,}\dot{v,}\dot{d}]}^{T}$$ (3)

where u and v represent the *X* (azimuth) and *Y* (LaserID) values of the center of the target; d is DbLV and $\dot{u,}\dot{v,}\dot{d}$ are the rates of corresponding variables, representing the changes of u, v, and d respectively. The next state at time k(${\hat{x}}_{k}$) is predicted based on the current state ${\hat{x}}_{k-1}$ using the following equation:


$${\hat{x}}_{k}=F_{k}{\hat{x}}_{k-1}$$ (4)

where $F_{k}$ is the prediction matrix. The time between two successive frames depends on the frequency of LiDAR rotation (0.1s for 10 Hz). $F_{k}$ is set by following because the movement of vehicle can be regarded as a linear constant velocity model during such a short time. $F_{k}$ is set in this way where rates are only related to themselves while u, v and d is the results of combination of themselves and their corresponding rates.


$$F_{k}=\left[\begin{array}{c} 1,0,0,1,0,0\\ 0,1,0,0,1,0\\ 0,0,1,0,0,1\\ 0,0,0,1,0,0\\ 0,0,0,0,1,0\\ 0,0,0,0,0,1 \end{array}\right]\;H_{k}=\left[\begin{array}{c} 1,0,0,0,0,0\\ 0,1,0,0,0,0\\ 0,0,1,0,0,0 \end{array}\right]$$

The detection at time k is further refined using (5) with a measurement matrix $H_{k}$ since the variables u,v,d get updated every frame and the rates of corresponding variables are unknown.


$$z_{k}{{=H}_{k}x}_{k}+R_{k}$$ (5)

where $z_{k}$ is the measurement value while $R_{k}$ is the measure uncertainty. $x_{k}$ represents the true state of the system, but it is typically unknown. The observed value ($z_{k}$) is merged with the estimated value (${\hat{x}}_{k}$) according to (6) to get the best estimated state of the vehicle:


$${\hat{x}}_{k}^{\prime }{=\hat{x}}_{k}+K^{\prime }(z_{k}-\;{H_{k}\hat{x}}_{k})$$ (6)

likewise, the covariance matrix $P_{k}=F_{k}P_{k-1}F_{k}^{T}$ [46], which indicates correlation between each variable in ${\hat{x}}_{k},$ is updated by:


$$P_{k}^{\prime }=P_{k}-K^{\prime }\;H_{k}P_{k}$$ (7)

where $K^{\prime }$ is the term after Kalman gain K take off $H_{k}:$


$$K^{\prime }=P_{k}H_{k}^{T}{\left(H_{k}P_{k}H_{k}^{T}+R_{k}\right)}^{-1}$$ (8)

#### 2.3.3 Data association using hungarian algorithm.

The Hungarian Algorithm associates the current predictions to the new detections using an assignment cost matrix. Each cell in the cost matrix represents the relation between the corresponding objects. The u,v and d values of each vehicle are estimated by predicting its new location in the next frame using the information from the current frame. To assign the prediction to the corresponding detection in the next frame, an index is developed to calculate the gap between the two:


$$Index=\hat{u}+\hat{v}+\hat{d}$$ (9)

where $\hat{u}$, $\hat{v}$, and $\hat{d}$ are normalized *u*, *v*, and *d* values respectively. Then, the assignment cost matrix is computed as the Index difference by pairing each detection with all the predictions from the existing targets based on the global nearest neighbor:


$${Index}_{diff}=\left|{\hat{u}}_{k}-{\hat{u}}_{k-1}\right|+\left|{\hat{v}}_{k}-{\hat{v}}_{k-1}\right|+|{\hat{d}}_{k}-{\hat{d}}_{k-1}|$$ (10)

**Fig 3:**
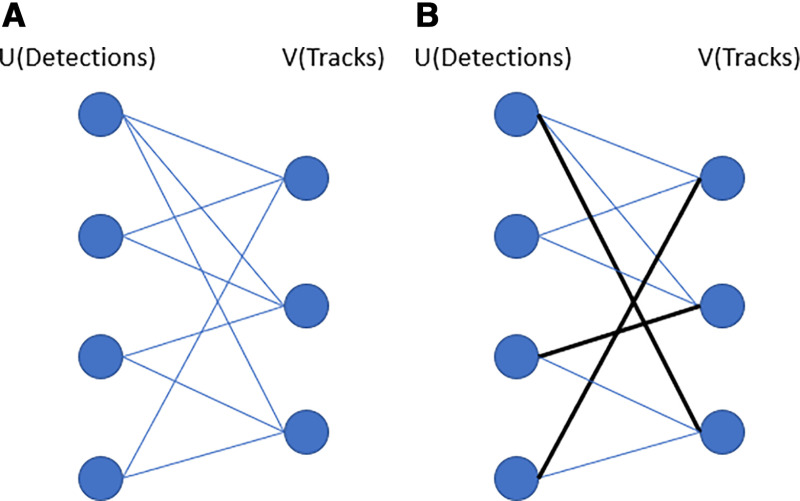
Assignment of Hungarian Algorithm.

The Hungarian algorithm is used to solve (2) in order to get a globally optimal answer. Suppose there are *m* detections of vehicles and *n* tracks of vehicles, the Hungarian algorithm assigns each track to every measurement such that the total cost is minimized. In this study, this cost is represented by the proposed index. Consider G=(U,  V, E) be a bipartite and weighted graph as shown in Fig 3, the black lines in right hand picture are the assignment of Hungarian algorithm from all possible connections (blue lines).

Each edge *e* connects two nodes, and every node *v* is covered exactly once following inequality:


$$\omega \left(M^{\prime }\right)=\sum_{e\in E}\omega (e)\leq \sum_{e\in E}\left(l\left(e_{d}\right)+l\left(e_{t}\right)\right)=\sum_{v\in V}l(v)$$ (11)

where $M^{\prime }$ is any perfect matching by a random assignment of vertices, $e_{d}$ and$\;e_{t}$ are the edges in detection and tracks respectively, and l(v) is a numeric label to node v. Now let M be a perfect match, then:


$$\omega (M)=\sum_{e\in E}\omega (e)=\sum_{v\in V}l(v)$$ (12)

when $\omega \left(M^{\prime }\right)$ ≤ ω(M) and M is optimal.

The core steps of the Hungarian algorithm are described as follows:

- For each row of the matrix, find the smallest element and subtract it from every element in its row.
- Repeat step 1 for all columns.
- Cover all zeros in the matrix using minimum number of horizontal and vertical lines.
- Test for Optimality: If the minimum number of covering lines is *n* (the number of new detections in our case), an optimal assignment is possible then the process is finished. On the other hand, if the number of lines is less than *n* then it means that the optimal assignment hasn’t been found yet, and step 5 must be carried out.
- Determine the smallest entry not covered by any line. Subtract this entry from each uncovered row, and then add it to each covered column. Return to step 3.

A threshold ${Index}_{\max}$ is used to reject the assignments where the nearest neighbor is larger than ${Index}_{\max}$. Suitable threshold value can be selected based on the specific traffic conditions. For example, given that the speed limit is 70 km/h, d should not be larger than 2m because the vehicle is not allowed to move that fast, considering 2m/0.1s equals 72 km/h. Similarly, the threshold for u can also be calculated by the traffic conditions while v wouldn’t change much as the height of vehicle or the level of terrain wouldn’t change much.

When compared to traditional bounding box-based tracking, the combination of “Track by Point” and “SORT” offers several advantages. Track by point tracks specific key points on an object, enabling better handling of appearance changes, occlusions, and object interactions. Track by point can be computationally more efficient compared to tracking entire bounding boxes, especially in situations with multiple objects. Bounding box-based tracking involves processing larger amounts of data, which can lead to higher computational requirements. Track by point also excels at handling occlusions since it can still track visible key points even when parts of an object are hidden.

## 3. Result and discussion

**Fig 4:**
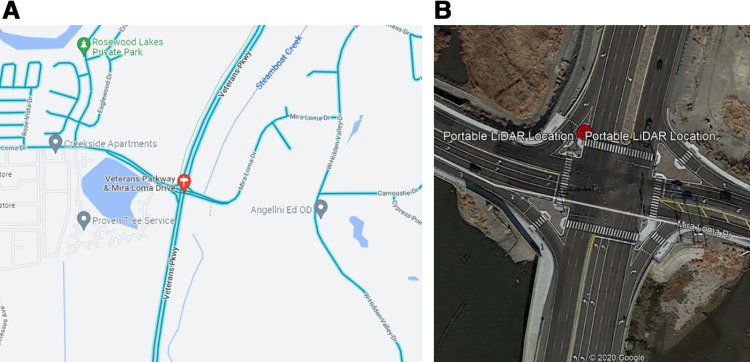
Location of LiDAR at Veterans & Mira Loma intersection. (a) The site location of Veterans & Mira Loma intersection. (b) The location of Lidar at the intersection.

Two datasets were used to verify the algorithm. The dataset collected from Reno, Nevada includes two intersections, namely Veterans & Mira Loma intersection, and 4th & Virginia intersection. The other dataset is located at Quaker Avenue and 50^th^ Street, Lubbock, Texas. A portable roadside LiDAR (VLP-32 of Velodyne, 10 Hz) was equipped at the corner of each intersection in order not to interrupt the traffic. Fig 4 shows an example of the LiDAR location at Veterans & Mira Loma intersection.

The proposed algorithm’s robustness was evaluated using the Reno datasets, which involved comparing the performance of using bounding boxes versus the proposed index, assessing the influence of data size on running time, and, notably, evaluating the impact of thresholds on the tracking accuracy. In addition, the limitations and potential future work for the proposed algorithm were illustrated using the Lubbock dataset, which consisted of 2000 frames. Despite the fact that the Lubbock dataset has a higher volume of traffic, the performance analysis was conducted using the Reno datasets due to its two intersections (one from downtown area), allowing for a more detailed evaluation of the algorithm’s performance. All data and algorithms were tested using an Intel i7 machine with 3.70 GHz processor and 16 GB memory.

### 3.1 Data preprocessing

**Fig 5:**
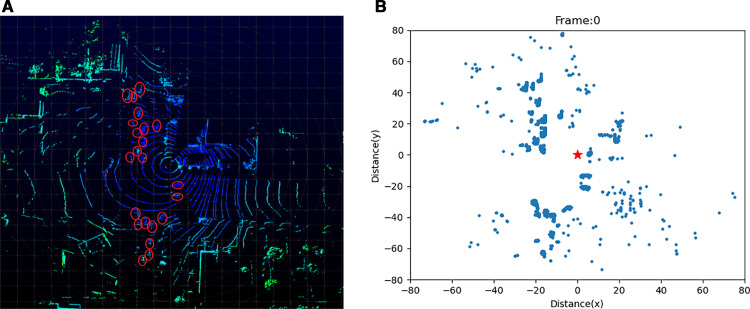
Demonstration of the Results of Background Filtering. (a) Raw data without background filtering. (b) Processed data after background filtering. *The red circles in the image on the left-hand side highlight the objects (vehicles) while the red star on the right-hand side indicates the location of the LiDAR, and the same applies to the images below.*

As aforementioned, background filtering and object clustering are processed before tracking. Fig 5 demonstrates the difference before and after background filtering.

Fig 5(a) is the raw data collected from Quaker Avenue & 50^th^ Street, Lubbock, Fig 5(b) is the results after background filtering. 3320 data points were retained after background filtering from 43769 raw data points, which means 92% points were removed. This significant reduction in data points can help expedite the following data processing steps. The proposed background filtering method requires 5 min to process 2000 frames, which is 6 times faster than the 3D-DSF algorithm.

From Fig 5(b), it is evident that some background points remain. However, the background filtering serves its purpose effectively because: 1) the majority of background points have been filtered out, and further filtering would diminish the points of interest; and 2) the subsequent clustering step will further contribute to the filtering of remaining background points.

**Fig 6:**
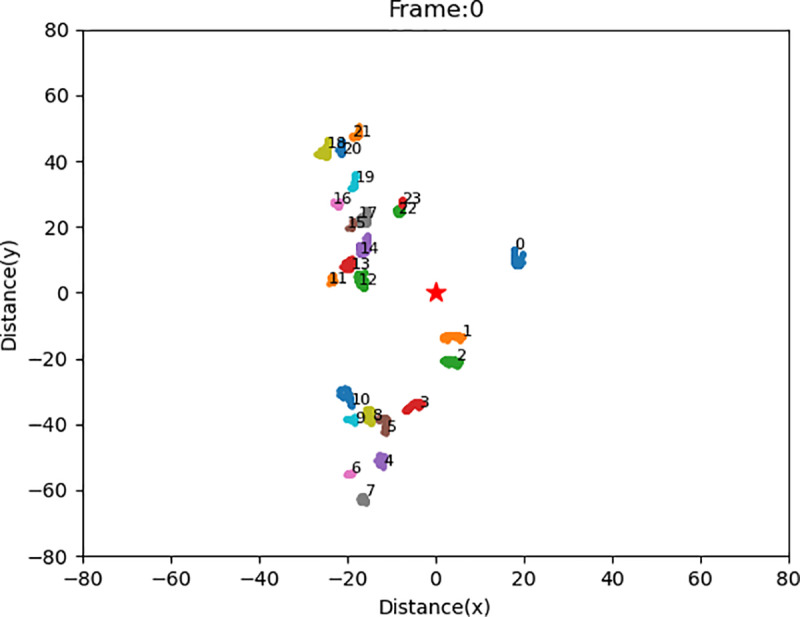
Demonstration of the Result of Clustering.

Fig 6 shows the results of clustering at the intersection of Quaker Avenue & 50^th^ Street, Lubbock. At a speed of 0.011s per frame with a mean accuracy of 0.9668, the clustering algorithm lays a solid ground for real-world applications.

### 3.2 Tracking

The Veterans & Mira Loma intersection serves as an example to illustrate the setting of thresholds and to assess performance metrics and runtime compared to the bounding box approach. Following this, data from the 4th & Virginia intersection is analyzed to further validate the durability of these thresholds. The 4th & Virginia intersection is situated in a downtown area that includes pedestrians, leading to an increase in the dataset’s size. Conversely, the Veterans & Mira Loma intersection has lower traffic volume and no pedestrian activity during data collection.

#### 3.2.1 Determination of threshold.

When using LiDAR data with the SORT algorithm, several parameters require careful adjustment to suit the specific data structure and motion dynamics. The state vector is redefined as Eq (3). $T_{lost}$ is a parameter in SORT to control when the algorithm decides to abandon the missed objects that are tracked. $T_{lost}$ is set to 1 indicating that tracking for the target is terminated if they are not detected for 1 frame because the frame-to frame tracking is concerned with real-time application, which requires a high resolution of detection. Additionally, unique identities are created or destroyed accordingly when the vehicles enter or leave the observation. Consequently, the matching parameters have been adapted to utilize a composite ${Index}_{diff}$ that integrates u,v,d, reflecting the spatial and angular characteristics of LiDAR data. According to our study performed at a similar site, Veterans Pkwy & E Greg St intersection, average speed was about 15 MPH at the stop bar for northbound vehicles that slowed but did not stop, so ${Index}_{\max}$ for u,v,d are set to 15, 2, and 2 respectively according to the traffic condition. Before comparing the performances of bounding box and the proposed Index method, the determination of the threshold for ${Index}_{\max}$ was examined by taking random examples from the dataset as shown in Table 2.

**Table 2:** The Mean and Standard Deviation for Variables Used in the Index.

	$m_{1}\pm \sigma_{1}$	$m_{2}\pm \sigma_{2}$	$m_{3}\pm \sigma_{3}$	Threshold
$\left|u_{t+1}-u_{t}\right|$	1.375±2.0812	8.3960±2.2561	7.5455±2.332	15
$\left|v_{t+1}-v_{t}\right|$	0.125±0.3378	0.1887±0.395	0.1818±0.3892	2
$\left|d_{t+1}-d_{t}\right|$	0.3712±0.3051	0.0906±0.0826	**0.8187** ± **0.55**	2

m*: mean value;* σ*: standard deviation.*

Speed significantly influences the thresholds for u and d. Faster speeds result in larger changes in u and d, necessitating more dynamic thresholds. To address this, a single threshold calculated by m+3σ is able to cover the most cases (99.73% statistically) as shown in Table 2. Since ${Index}_{\max}$ is the aggregation of all thresholds, ${Index}_{\max}$ is still acceptable if one of thresholds is beyond the limit of 3σ (The bold in Table 2). $m_{1}$ to $m_{3}$ represents the mean value for different scenarios. This approach ensures robustness against rapid motion while minimizing mismatches, offering a balance between sensitivity and stability in tracking performance.

#### 3.2.2 Metrics and evaluation.

The ground truth of vehicle numbers counted manually in a total of 500 frames of Reno datasets is 2170. The following evaluation metrics are applied to measure the performance. It is notable that multi-object tracking precision (MOTP) isn’t assessed because it measures the precision of bounding box.

- MOTA(↑): multi-object tracking accuracy.
- FP(↓): number of false detections.
- FN(↓): number of missed detections.
- ID sw(↓): number of times an ID switches.

Equation (13) shows the relation of the metrics while T is the ground truth.


$$MOTA=1-\frac{FP+FN+ID\;sw}{T}$$ (13)

Background filtering was employed to remove all non-vehicle points from the dataset, which could be the reason for the rare FP observed when utilizing both the bounding box method and the proposed index, as demonstrated in Table 3. For evaluation measures with (↑), higher scores denote better performance; while for evaluation measures with (↓), lower scores denote better performance. The results were evaluated for consecutive 500 frames. Although there are only a few increases regarding MOTA and FN, ID sw decreases nearly by 40% from 28 to 17, which significantly benefits tracking vehicles.

**Table 3:** The Performance Comparison between bounding box and the proposed index for tracking.

	MOTA(↑)	FP(↓)	FN(↓)	ID sw(↓)
Bounding box	0.9086	15	155	28
Proposed index	0.9253	12	**133**	**17**

**Fig 7:**
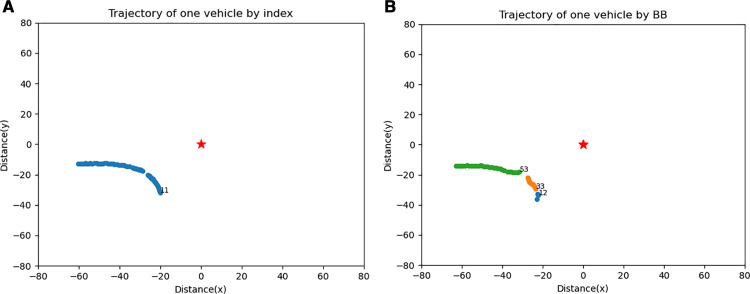
Demonstration of One Vehicle Tracked by Different Methods. (a) Tracking result of proposed method. (b) Tracking result of bounding box.

Fig 7 is an example showing how the tracking results of a single vehicle differ based on the tracking method used. The trajectory in a is tracked using the proposed index and has a unique tracking ID throughout its entire lifespan. Additionally, it exhibits a smooth trajectory that is more consistent with real-world movements as compared to the trajectory in b, which is tracked using a bounding box method.

#### 3.2.3 Runtime.

When comparing the computation time for the two algorithms used in this study, an improvement of 23% was observed with the proposed method compared to the bounding box method for tracking. From the experiment, a tracking time of 0.24s or 2086.4 was obtained when the bounding box method was used compared to 0.195s or 2566.4 FPS with the proposed method.

#### 3.2.4 Test for threshold.

To further test the robustness of the proposed index, and the impact of thresholds on tracking accuracy, 4th & Virginia intersection in downtown was conducted with various threshold values of 0.05, 0.1, and 0.2 after standardization. The results were compared to using bounding box in Table 4. The proposed index gains a better tracking accuracy than using the bounding box method (0.81 vs 0.79). The running time is provided in Table 5 as 792 FPS to 563 FPS

**Table 4.** The Comparison of MOTA for Different Thresholds.

Frame	Threshold = 0.05			Threshold = 0.1			Threshold = 0.2			Bounding Box		
	FP	FN	ID sw	FP	FN	ID sw	FP	FN	ID sw	FP	FN	ID sw
1-100	0	222	4	0	211	1	0	209	4	0	202	1
101-200	0	266	29	0	199	14	1	160	12	0	180	9
201-300	2	210	29	3	91	17	3	62	16	0	92	12
301-400	0	104	14	4	37	8	6	18	7	5	23	6
401-500	8	44	6	7	28	3	9	13	3	7	40	3
MOTA	0.666192171			0.778291815			**0.813879004**			0.793594306		

**Table 5.** Summary of Dataset Scenario and Performance.

	Veterans & Mira Loma		4th & Virginia	
	Index	Bounding box	Index	Bounding box
Speed Limit	55 & 30 MPH		25 & 25 MPH	
Frame duration	500(2170 vehicles)		500(2810 vehicles)	
Traffic composition	cars		Semi-truck, pedestrians, cars	
MOTA	**0.9253**	0.9086	**0.8139**	0.7936
Running Time(FPS)	**2566**	2086	**792**	563

**Fig 8.**
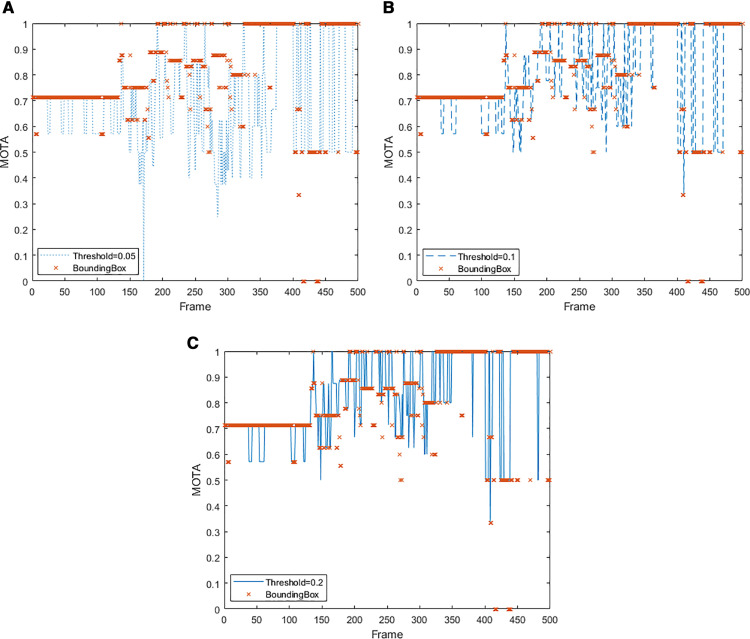
Comparison of MOTA for Each Frame. (a) BB VS 0.05 threshold, (b) BB VS 0.1 threshold, (c) BB VS 0.2 threshold.

It is notable that due to occlusion by the semi-truck and lower detection accuracy, ID sw in this case does not show an improvement. Fig 8 compares the MOTA between each threshold and bounding box for each frame at different thresholds. The tracking ac-curacy improves significantly when the threshold changes from 0.05 to 0.1. However, the improvement is much less when the threshold changes from 0.1 to 0.2 or from 0.05 to 0.1 even though the MOTA reaches its peak (0.81) at threshold = 0.2. From Table 5 and Fig 8, although the increased threshold would benefit the tracking accuracy, a suitable threshold is still expected due to the following reasons: 1) the large threshold could have the error that the closed vehicles can be identified as one object. 2) the margin of profit by increasing the threshold is diminishing as the accuracy only gets slight improvement from 0.78 to 0.81 when the threshold changes from 0.1 to 0.2.

Table 5 summarizes the tracking performance at the two study intersections. The proposed index not only achieves higher accuracy but also demonstrates a runtime advantage, achieving 792 FPS compared to 563 FPS when the bounding box method is used. The reduction in FPS is expected due to the increased data size from higher traffic volumes. However, the improvement in computation time ensures the capability for real-time applications.

### 3.3 Limitations

The dataset from Quaker Avenue and 50th Street in Lubbock, which includes only vehicles (cars and trucks, averaging 25 vehicles per frame), was evaluated over 2000 frames. The results showed a performance of 603 FPS and a MOTA of 0.85, compared to 235 FPS and a MOTA of 0.75 when using the bounding box method.

**Fig 9:**
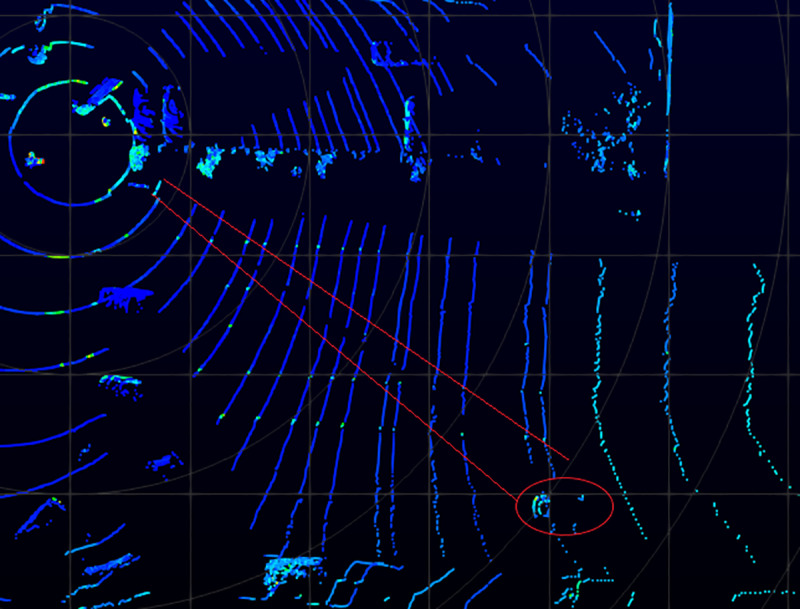
Raw data at Lubbock Intersection at Frame 53.

Per our knowledge, the occlusion is the main reason that causes a worse track accuracy. As shown in Fig 9, there is a barrier that covers the area between the LiDAR and objects (red circle). Although in the beginning, the shadow is narrow, it expands to large enough to make the whole object disappear as the distance away from LiDAR increases

**Fig 10:**
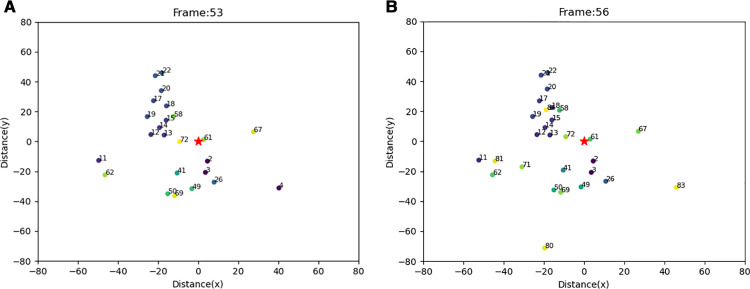
The Impact of Occlusion on Tracking Accuracy. (a) Tracking result of frame 53, (b) Tracking result of frame 56.

This could lead to ID switches in tracking, such as Vehicle 4 in frame 53 changing to Vehicle 83 in frame 56 (as seen in Fig 10). Even more critically, the object might not be detected at all.

To address such challenges, recent advancements in occlusion handling include the use of transformer-based tracking algorithms and multi-sensor fusion techniques. Transformer-based approaches employ attention mechanisms that help maintain consistent tracking even under partial or full occlusions, while multi-sensor fusion—integrating data from LiDAR, radar, and video—offers complementary information that can mitigate occlusion problems. Additionally, techniques such as adjusting the height of the LiDAR sensor can help minimize occlusion caused by large trucks or barriers, allowing for a more complete field of view, especially for small vehicles hidden behind larger objects.

## 4. Conclusion

The new tracking model demonstrates improved performance on two datasets, particularly in terms of execution speed, which is advantageous for real-time applications. While it may not significantly enhance overall accuracy, the reduction in ID switches aids in preserving the continuity of vehicle trajectories. However, the model’s dependency on specific traffic scenario thresholds limits its widespread application. Additionally, occlusions caused by any objects situated between the LiDAR and vehicles pose a significant issue, especially when the LiDAR is positioned at a complex intersection. Given that the portable Lidar is mounted at a height of 2.5 meters, adjusting the Lidar’s height and location might mitigate these occlusion issues. Future research could aim to develop a more accurate and universally applicable method.

This paper presents a novel tracking index inspired by tracking by points, instead of the conventional bounding box method, to facilitate frame-to-frame vehicle tracking utilizing 2D LiDAR data within the SORT tracking framework. An experimental study was conducted to evaluate the proposed methodology and compare it with the state-of-the-art tracking method that uses the bounding box approach. The results showed that the proposed index reduces the number of ID switches when the detection is of sufficient quality, thereby aiding in consistent vehicle tracking. Moreover, the proposed approach significantly reduces the running time, enabling real-time assignment without sacrificing accuracy. Additionally, the newly proposed background filtering method yielded better accuracy, as evidenced by the reduced number of false positives during detection. However, to achieve enhanced tracking in the presence of occlusions, better detection methods, not only in terms of algorithms but also LiDAR installations, are required.
